# Full title: High glucose protects mesenchymal stem cells from metformin-induced apoptosis through the AMPK-mediated mTOR pathway

**DOI:** 10.1038/s41598-019-54291-y

**Published:** 2019-11-28

**Authors:** Xiao He, Yi Yang, Meng-Wei Yao, Ting-ting Ren, Wei Guo, Ling Li, Xiang Xu

**Affiliations:** 1 Department of Stem Cell and Regenerative Medicine, State Key laboratory of Trauma, Burn and Combined Injury, Daping Hospital, Army Military Medical University, Chongqing, P.R. China; 2 Central Laboratory, State Key laboratory of Trauma, Burn and Combined Injury, Daping Hospital, Army Military Medical University, Chongqing, P.R. China; 3 Department of Rheumatology and Clinical Immunology, Daping Hospital, Army Military Medical University, Chongqing, P.R. China; 4 Department Biochemistry and Molecular Biology, College of Basic Medical Sciences, Army Medical University, Chongqing, P.R. China; 5 Department of Histology and Embryology, Qingdao University Medical College, Qingdao, Shandong P.R. China; 6 PLA Rocket Force Characteristic Medical Center, Beijing, P.R. China

**Keywords:** Apoptosis, Diabetes complications

## Abstract

Micro- and macro-vascular events are directly associated with hyperglycemia in patients with type 2 diabetes mellitus (T_2_DM), but whether intensive glucose control decreases the risk of diabetic cardiovascular complications remains uncertain. Many studies have confirmed that impaired quality and quantity of mesenchymal stem cells (MSCs) plays a pathogenic role in diabetes. Our previous study found that the abundance of circulating MSCs was significantly decreased in patients with T_2_DM, which was correlated with the progression of diabetic complications. In addition, metformin-induced MSC apoptosis is one of the reasons for the decreased quantity of endogenous or exogenous MSCs during intensive glucose control. However, the role of glucose in metformin-induced MSC apoptosis during intensive glucose control in T_2_DM remains unknown. In this study, we found that metformin induces MSC apoptosis during intensive glucose control, while high glucose (standard glucose control) could significantly reverse its adverse effect in an AMPK-mTOR pathway dependent manner. Thus, our results indicate that the poorer clinical benefit of the intensive glucose control strategy may be related to an adverse effect due to metformin-induced MSC apoptosis during intensive glucose control therapy in patients with T_2_DM.

## Introduction

Hyperglycemia is a hallmark of diabetes mellitus. Epidemiologic studies indicate that cardiovascular disease is a major cause of death in patients with type II diabetes mellitus (T_2_DM); however, the incidence of diabetic cardiovascular complications is directly associated with the degree of hyperglycemia as measured by the plasma glucose or glycated hemoglobin level. After adjusting for other risk factors, an increase of 1% in the glycated hemoglobin level is positively linked to an increase of 18% in the risk of cardiovascular events^1^, which, in turn, increases the risk of death by 12% to 14%^2,3^. The tight connection between the glycated hemoglobin level and cardiovascular risks led to the development of a therapeutic strategy to lower the glycated hemoglobin level to a normal level, namely, intensive glucose control. However, the intensive glucose control theory was challenged by recent studies aiming to determine the effects of intensive glucose lowering in T_2_DM. The UKPDS study reported that intensive glucose control substantially decreases the risk of microvascular complications but not macrovascular diseases in T_2_DM^4^. The ADVANCE study reported that intensive glucose control has no effects on macrovascular events, death due to cardiovascular causes, or death due to any cause but is associated with a relative reduction in nephropathy^5^. The VADT study found that intensive glucose control has no significant effect on the rates of major cardiovascular events, death, or microvascular complications^6^. Even worse, the ACCORD study reported that intensive glucose control targeting normal glycated hemoglobin levels (i.e., below 6.0%) actually increased mortality and did not significantly reduce major cardiovascular events compared with standard glucose control in high-risk patients^7,8^. Although these results cannot negate the importance of intensive glucose control therapy in the management of T_2_DM, the degree, strategies and methods of glucose control and the underlying mechanisms and reasons need to be reconsidered.

Due to its multipotent differentiation potential and immunomodulatory capacities, mesenchymal stem cell (MSC)-based regenerative therapy is currently considered an alternative approach to the treatment of many diseases, including diabetes and its complications^9^. To clearly define and verify MSCs, the International Society for Cellular Therapy (ISCT) proposed minimal criteria for the definition of human MSCs^10^. While there are no minimal criteria established for the identification of mouse MSCs, mouse MSCs are generally characterized by the positive expression of CD29, CD44, CD73, CD105, CD106, and Sca-1 and the negative expression of hematopoietic and the endothelial markers CD45, CD11b, and CD31^11–13^.

Metformin has been considered a first-line anti-diabetic drug for many years, and AMP-activated protein kinase (AMPK), which is a cellular metabolic sensor, is a very well characterized target of metformin. Previous studies have demonstrated that AMPK could directly and/or indirectly inhibit mammalian target of rapamycin complex 1 (mTORC1) activity by phosphorylating TSC2 on Ser1387^14^. AMPK also directly phosphorylates Raptor, which is a subunit of the mTORC1 complex, on Ser722 and Ser792, resulting in the full suppression of mTORC1^15,16^. Mammalian target of rapamycin (mTOR), especially mTORC1, could regulate anabolic processes, i.e., the synthesis of proteins, lipids and nucleic acids through S6 kinase (S6K), 4E-binding protein (4E-BP1), etc.^17^. Based on the above mechanism, AMPK alters cell metabolism by inhibiting mTORC1, which enhances the rate of catabolic pathways and attenuates the rate of anabolic pathways. AMPK also directly affects acetyl-CoA carboxylase (ACC), which is a key regulatory enzyme of fatty acids^18^. Recently, our group found a significant decrease in the abundance of circulating MSCs, which was correlated with complications in patients with T_2_DM^19^. In addition, we found the unexpected adverse effect that metformin, as a first-line agent used to treat T_2_DM and a basic glucose-lowering drug used during intensive glucose control, significantly induced MSC apoptosis and damped their therapeutic efficacy in infarcted myocardium^20^. However, the role of glucose during metformin-induced MSC apoptosis in diabetes remains unknown.

In this study, we tested the effect of different concentrations of glucose on metformin-induced MSC apoptosis and investigated the underlying mechanism of the inhibitory effect of glucose on metformin-induced MSC apoptosis. We found that glucose could modulate metformin-induced MSC apoptosis via the AMPK/mTOR pathway, i.e., high glucose (standard glucose control) inhibits apoptosis, while low glucose (intensive glucose control) facilitates apoptosis.

## Research design and methods

### Cells

The human umbilical cords derived MSCs (hUC-MSCs) were provided by Kangcell Biotechnology Company (Chongqing, China) and isolated and cultured as previously described^20^. The siRNAs were synthesized by RiboBio Company (Guangzhou, China). The oligonucleotide transfection was conducted by using Lipofectamine RNAiMAX transfection reagent (Life Technologies, USA) following the protocol recommended by the manufacturer. After 48 h of transfection, the cells were collected and used for further investigations. According to the results of the glucose levels detected by high-performance liquid chromatography (HPLC) in cell culture medium every 24 h during the experiment, the corresponding glucose level was added to the medium to achieve the initial culture concentration. After incubation in metformin (2 mM) with or without glucose (6.1 mM, 10 mM, 15 mM, or 30 mM) and/or siAMPK (50 nM)/siControl (50 nM) or Compound C (1 μM) at 24 h, 48 h and 72 h in α-minimum Eagle’s medium (α-MEM) (5.5 mM) basic medium, the MSCs were washed with PBS and stained using an Annexin V–FITC and propidium iodide (PI) apoptosis kit (KeyGen Biotech, China) according to the manufacturer’s instructions to quantify apoptosis. The stained cells were collected and analyzed by flow cytometry (NovoCyteTM, ACEA Biosciences, USA), and at least 10^5^ events were analyzed. The data were analyzed by using NovoExpress V.1.2.1 software (NovoCyteTM, ACEA Biosciences, USA).

### Mice

B6.BKS(D)-*Lepr*^db^/J mice (Jackson Laboratory, Bar Harbor, ME, USA) were bred at the Experimental Animal Center of the Army Medical Center, Army Military Medical University. Based on the normalization to the body surface area, the human metformin dose of 20 mg/kg/day orally translates to a mouse equivalent dose of 250 mg/kg/day^21^. In this study, 8- to 12-week old male diabetic mice were treated with saline, metformin (250 mg/kg/d, i.g., Sigma), metformin + compound C (0.1 mg/kg/d, i.g., Sigma), or metformin + glucose (1000 mg/kg/d, i.g., Sigma) by oral gavage for 4 weeks (n = 5 per group). Following the 4-week treatment period, all diabetic mice were sacrificed to isolate the bone marrow MSCs for a flow cytometry assay. Animal care and all experimental procedures were performed in accordance with the approved protocols and animal welfare regulations of the Animal Care and Use Committee of the Army Military Medical University.

### Co-immunoprecipitation and western blotting

After the treatment with metformin (2 mM), glucose (15 mM) and/or siAMPK (50 nM)/siControl (50 nM) or Compound C (1 μM) at 24 h or 48 h, the cells were lysed in ice-cold NP-40 buffer containing protease inhibitor (1 mg/ml aprotinin, 1 mg/ml leupeptin, 1 mg/ml pepstatin, 1 mM sodium orthovanadate, and 1 mM phenylmethylsulfonyl fluoride). The whole-cell lysates were incubated with protein A/G Sepharose beads (Roche) for 2 h at 4 °C, and the beads were discarded to eliminate non-specific binding. Then, the supernatants were incubated overnight with a Bcl-xl antibody (Cell Signaling Technology, Danvers, USA) at 4 °C, followed by incubation with protein A/G Sepharose beads for another 2 h at 4 °C. Then, western blotting was performed with the indicated antibodies according to standard protocols as previously described^20^. The primary antibodies, including p-AMPK (T172) (#50081), phospho-Acetyl-CoA carboxylase (p-ACC) (#11818), phospho-Tuberin/TSC2 (p-TSC2) (Ser1387) (#23402), phospho-Raptor (p-Raptor) (Ser792) (#2083), p-mTOR (S2448) (#5536), phospho-p70 S6 kinase (p-S6K1) (Thr389) (#97596), p-ULK1 (Ser757) (#14202), p-4E-BP1 (Thr70) (#9455), cleaved Caspase-3 (Cl-Caspase3) (Asp175) (#9664), p-Akt (Ser473) (#4060), p-Akt (Thr308) (#13038), RagB (#8150), LC3B (#3868), β-Actin (#3700) were all purchased from Cell Signaling Technology, Danvers, USA.

### Real-time cell proliferation monitoring

The MSCs were seeded at densities of 5.5 × 10^3^ cells/well in an E-plate 16 (ACEA Biosciences, San Diego, USA) containing 100 µL culture medium per well and monitored on an xCELLigence Real-Time Cell Analyzer Dual Plate (RTCA DP, USA) instrument (ACEA Biosciences, USA). When the cells entered the log phase, 2 mM metformin, 2 mM metformin + 1 μM compound C, or 2 mM metformin + glucose solution (6.1 mM, 10.0 mM, 15 mM and 30 mM) were added in α-MEM (5% FBS, 2 mM L-Glutamine). The cells were treated for 7 days and incubated at 37 °C in a 5% CO_2_ atmosphere. RTCA software v. 1.2.1 was used to record the cell index (CI), and all experiments were repeated at least 3 times.

### Definitions of different types of glucose levels

By referencing the normal adult blood glucose level and normal glucose concentration in the cell culture medium, we defined the glucose levels as follows: Normal glucose =6.1 mM (110 mg/dL), Low glucose =< 6.1 mM (110 mg/dL); High glucose = 6.1 mM (110 mg/dL) to 30 mM (540 mg/dL).

### Statistical analysis

All experiments were repeated at least three times. The data are presented as the means ± standard deviation (SD). The statistical analysis (one-way analysis of variance (ANOVA) followed by a Bonferroni test) was performed using SPSS 13.0 software (USA); p < 0.05 was considered statistically significant.

## Results

### Glucose levels impact metformin-induced MSC apoptosis

**Figure 1:**
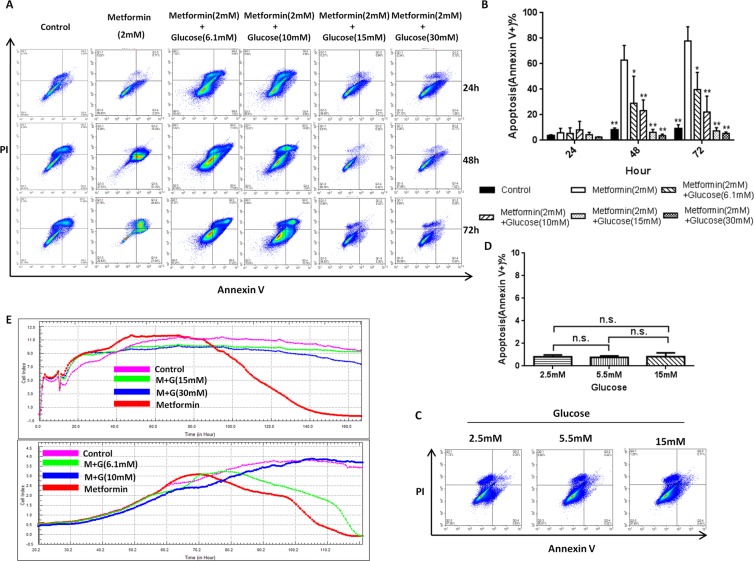
Glucose modulates MSC resistance to metformin-induced apoptosis *in vitro*. (**A,B**) The effect of different concentrations of glucose on metformin-induced hUC-MSC apoptosis (Annexin V+) at 24 h, 48 h and 72 h. Bars in B represent the mean ± SD (n = 3 per group). The concentration of glucose in the control and metformin groups is 5.5 mM. *p < 0.05, **p < 0.01 compared with the metformin (2 mM) group at each time by a one-way ANOVA. (**C,D**) The ratio of hUC-MSC apoptosis (Annexin V+) induced by different concentrations of glucose at 72 h. Bars in D represent the mean ± SD (n = 3 per group). n.s. = no significance by a one-way ANOVA. E, Real-Time Cellular Analysis (RTCA) of different concentrations of glucose on metformin-induced hUC-MSC apoptosis; the glucose concentration in the control group is 5.5 mM.

Since intensive glucose control does not benefit diabetic patients, is it possible that low glucose levels (intensive glucose control) also participate in the adverse effects of metformin? To address this question, the effects of different concentrations of glucose on metformin-induced hUC-MSC apoptosis were evaluated. Surprisingly, a significant reduction in the number of apoptotic cells was detected by flow cytometry after 48 h of the glucose treatment from 66% in the low glucose group (100 mg/dL glucose) to 1% in the high glucose group (270 mg/dL glucose) (Fig. 1A,B). In addition, an inverse relationship was seen between the increasing glucose concentrations and the degree of metformin-induced apoptosis from 48–72 h (Fig. 1B). Interestingly, after exposure to only low glucose, we did not detect a significant change in the MSC survival rate (Fig. 1C,D), suggesting that a low glucose level facilitates metformin in inducing, but not directly causes, MSC apoptosis. Meanwhile, high glucose alone has no effect on MSC apoptosis (Fig. 1C,D). In support of this result, the Real-Time Cellular Analysis (RTCA) also showed increased apoptosis in the hUC-MSCs at the lower glucose concentrations in the metformin group (5.5 mM glucose) and normal fasting blood glucose group (6.1 mM glucose), while high glucose showed a protective effect on metformin-induced apoptosis (Fig. 1E). These results indicate that different glucose levels affect metformin-induced MSC apoptosis in a dose-dependent manner.

### High glucose inhibited metformin-induced MSC apoptosis in an AMPK-mTOR-S6K1- Bad-dependent manner

**Figure 2:**
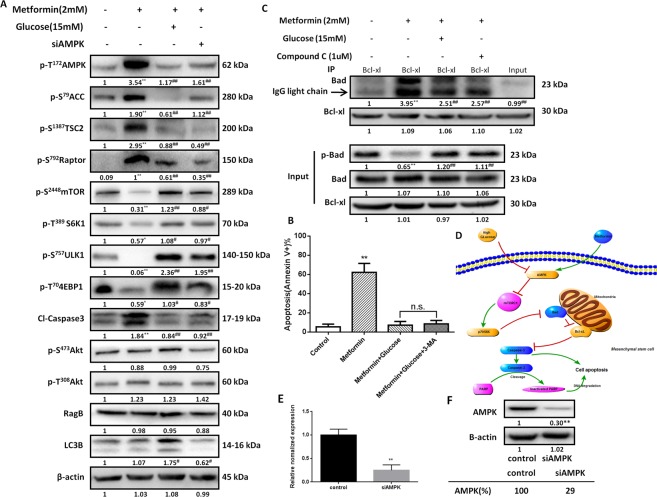
Glucose modulates MSC resistance to metformin-induced apoptosis in an AMPK-mTOR-S6K1-Bad-dependent manner. (**A**) The expression of cleaved caspase-3, AMPK and its downstream markers, and mTOR and its downstream markers in hUC-MSCs treated with metformin (2 mM) with or without glucose (15 mM) or siAMPK as determined by western blotting. β-actin was used as a loading control. The numbers under each blot represent the relative quantification of the band density, which was calculated as the ratio of the band density each group to the band density of the control group or the metformin group. The average grayscale intensities of the control bands are denoted as “1”, and the data are presented as the means. *p < 0.05, **p < 0.01, versus the control group, by one-way ANOVA. ^#^p < 0.05, ^##^p < 0.01, versus the metformin group, by one-way ANOVA. (**B**) After the treatment with metformin (2 mM), glucose (15 mM) and 3-MA (5 mM), the apoptosis (Annexin V+) rates were analyzed by flow cytometry, n = 5 per group. **p < 0.01, versus the control group, by one-way ANOVA. n.s. = no significance by one-way ANOVA. (**C**) hUC-MSCs were treated with metformin (2 mM) with or without glucose (15 mM) or siAMPK. After the treatment, the cell lysates were immunoprecipitated with an anti-Bcl-xL antibody and immunoblotted with an anti-Bad antibody. The presence of Bad and Bcl-xL in the lysates was examined. *p < 0.05, **p < 0.01, versus the control group, by one-way ANOVA. ^#^p < 0.05, ^##^p < 0.01, versus the metformin group, by one-way ANOVA. (**D**) The signaling pathway through which glucose modulates metformin-induced MSC apoptosis. Expression of AMPK in hUC-MSCs 24 h after the transient transfection of siAMPK at the E transcriptional (qRT-PCR) and F translational (western blot) levels. **p < 0.05 by t-test.

We previously reported that metformin induces MSC apoptosis depending on the AMPK/mTOR signaling pathway^20^. To explore whether the same pathway is associated with the protective effects of high glucose against metformin, we explored the serine/threonine kinase (Akt)- and AMPK-mTOR signaling pathways. Similar to the inhibitory effect of siAMPK (approximately 71% knockdown by siAMPK) (Fig. 2E,F), the high glucose treatment reversed the metformin-activated MSC AMPK, which suppressed mTOR and its downstream effector ribosomal protein S6 kinase beta-1 (S6K1) (Fig. 2A). In addition, the AMPK substrates, i.e., phospho-Acetyl-CoA Carboxylase (Ser79) (pACC-S79), phospho-Tuberin/TSC2 (Ser1387) (pTSC2-S1387) and phospho-Raptor (Ser792) (pRaptor-S792), activated by AMPK after the metformin treatment were inhibited by either the high glucose or siAMPK treatment (Fig. 2A). Furthermore, the mTORC1 substrates, i.e., phospho-4E-BP1 (Thr70) (p4EBP1-T70) and phospho-ULK1 (Ser757) (pULK1-S757), which were inhibited by phospho-AMPK after the metformin treatment, were also reversed by either the high glucose or siAMPK treatment (Fig. 2A). However, the glucose treatment group did not exhibit a significant change in Akt.

In addition to apoptosis-related caspase 3, the autophagy-related autophagy marker Light Chain 3B (LC3B) was involved. Compared to the metformin group, the LC3B level in the high glucose group was increased (Fig. 2A), but metformin-induced MSC apoptosis was not increased after the treatment with high glucose (15 mM) and/or an autophagy inhibitor (3-Methyladenine, 3-MA) (Fig. 2B). Additionally, both metformin and glucose did not change the level of Rag B (Fig. 2A), which is reportedly involved in mTOR inhibition by metformin^16^.

Previously, we reported that metformin induces MSC apoptosis in an AMPK-mTOR-S6k1-Bad-dependent manner. Therefore, does the inhibitory effect of high glucose on metformin-induced MSC apoptosis also depend on this pathway? Following the treatment with high glucose, the increased binding between Bad and Bcl-xL in the metformin group has significantly decreased, which was accompanied by a decrease in Bad phosphorylation (Fig. 2C). Taken together, these results demonstrate that high glucose protects MSCs from metformin-induced apoptosis in an AMPK-mTOR-S6k1-Bad-dependent manner (Fig. 2D).

### High glucose inhibited metformin-induced MSC apoptosis in diabetic mice (B6.BKS(D)-Leprdb/J)

**Figure 3:**
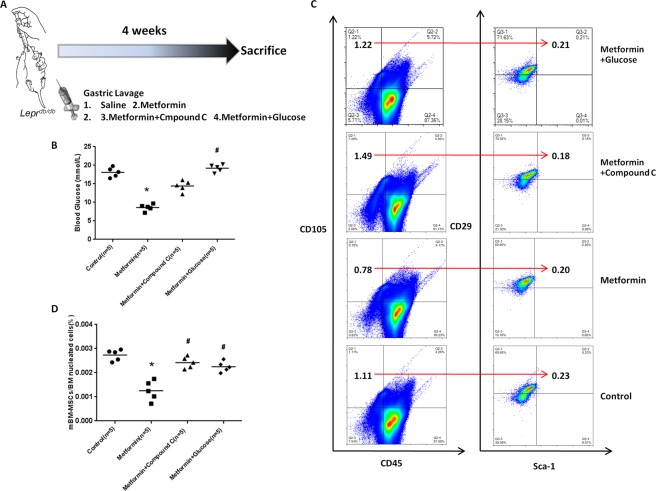
Glucose modulates MSC resistance to metformin-induced apoptosis *in vivo*. (**A**) Diabetic mice were treated with saline, metformin (250 mg/kg/d, i.g., n = 5), metformin and compound C (AMPK-inhibitor) (0.1 mg/kg/d, i.g., n = 5), or metformin and glucose (1000 mg/kg/d, i.g., n = 5) by oral gavage for 4 weeks; then, all mice were sacrificed to isolate the mouse bone marrow derived mesenchymal stem cells (mBMSCs) for a flow cytometry assay. (**B**) Blood glucose levels in different groups before sacrifice. *p < 0.05 vs. the control group; ^#^p < 0.05 vs. the metformin group by a one-way ANOVA. (**C,D**) Metformin treatment induced a significant decrease in mBMSCs compared with that in the saline group. Compared with the metformin group, glucose and compound C reduced the metformin-induced mBMSC decrease (CD45-CD105+ CD90+ Sca-1+). The direction of the arrow in the figure represents the proportion of the right cell group (CD29 + Sca-1+) in the cell group (CD45-CD105+) shown on the left. Lines in D represent the mean (n = 5 per group). *p < 0.05 vs. the control group; ^#^p < 0.05 vs. the metformin group by a one-way ANOVA. mBMSCs, mouse bone marrow mesenchymal stem cells.

The cell experiment data suggest that high-glucose could prevent metformin-induced MSC apoptosis. Is it possible that a high blood glucose level can prevent metformin-induced apoptosis in diabetic mice (B6.BKS(D)-Leprdb/J)? To address this question, we performed an *in vivo* experiment by treating diabetic mice (n = 5 per group) with either metformin or metformin combined with glucose to simulate “intensive glucose control” or “standard glucose control”, respectively. Considering the different surface markers of mouse MSCs in different tissue sources and previous studies^22–28^, we selected CD45-CD105 + CD90 + Sca-1 + as surface markers for the identification of mouse bone marrow-derived MSCs (mBM-MSC) by flow cytometry. As expected, the blood glucose levels in the standard glucose control group were significantly higher than those in the metformin group (Fig. 3A,B). After the treatment with saline, metformin (250 mg/kg/d), metformin + compound C (0.1 mg/kg/d), or metformin + glucose (1000 mg/kg/d) by gavage for 4 weeks, the diabetic mice in the metformin group showed a significantly lower level of blood glucose than the mice in the other groups (Fig. 3B). As expected, the metformin treatment induced a significant decrease in the mBM-MSCs in the diabetic mice compared with that observed in the saline group (p < 0.01) (Fig. 3C,D). Compared with the metformin group, high glucose and compound C significantly reduced the metformin-induced mBM-MSC decrease (CD45-CD105 + CD90 + Sca-1 + ) (p < 0.01) (Fig. 3C,D).

## Discussion

MSCs are multipotent cells with immunomodulation and tissue-repair capacities that are located in almost all tissues^29^. When a tissue is damaged, resident MSCs rapidly help recruit abundant MSCs from peripheral circulation to the injury site to participate in tissue repair and regeneration^30,31^. Notably, the efficacy of MSCs in tissue repair depends on their quality and quantity. Several studies have reported that an impaired quality of MSCs plays a pathogenic role in diabetes^32–34^. Consistent with these studies, we previously found that metformin-induced MSC apoptosis damped their therapeutic efficacy in infarcted myocardium in diabetic mice^20^. As metformin is a basic glucose-lowering drug during glucose control, there may be a relationship between glucose concentrations and metformin-induced MSC apoptosis. The results of our studies indicate that the glucose levels impact metformin-induced MSC apoptosis, and a clear inverse trend between increasing glucose concentrations and the degree of metformin-induced apoptosis was observed.

Subsequently, we analyzed the molecular mechanism by which high glucose inhibits metformin-induced MSC apoptosis. In addition to its glucose-lowering effect, recent studies have shown that metformin has an anti-tumor potential. An early finding supporting the anti-tumor effect of metformin was the observation that the drug inhibited breast cancer cells in an AMPK/mTOR dependent manner^35^. In addition, metformin targeted autophagy is mediated by AMPK activation and mTOR suppression through Raptor phosphorylation (Serine792)^36^. Moreover, metformin enhanced tamoxifen-mediated induction of apoptosis in breast cancer cells via the bax/bcl-2 apoptotic pathway and the AMPK/mTOR/p70S6K growth pathway^37^. Therefore, the AMPK/mTOR pathway may play a critical role enabling metformin to exhibit antitumor properties related to cancer development and anabolic process regulation, i.e., the synthesis of proteins, lipids and nucleic acids^38^. Consistent with these studies, our results demonstrated that metformin treatment activated AMPK and its downstream substrates, including pACC, pTSC2 and pRaptor, leading to the suppression of mTORC1 and its downstream substrates, including p4EBP1 and pULK1. However, similar to siAMPK treatment, the high glucose treatment significantly inhibited the metformin-activated AMPK-mTOR pathway. Additionally, only the high glucose treatment did not affect apoptosis in MSCs. In contrast, high glucose could significantly promote MSC proliferation via the PI3K/Akt/mTOR signal pathways^39^.

The AMPK cascade acts as a sensor of the cellular energy status and strongly responds to the cellular AMP:ATP ratio. Therefore, AMPK is activated by any stress that depletes cellular ATP, including oxidative stress, metabolic poisoning, nutrient deprivation, hypoxia, or biguanide drugs, such as metformin^40^. To avoid prolonged incubations with metformin in culture media, which could completely deplete glucose and create a massive energy crisis leading to cell death, we used HPLC to detect the glucose levels in the cell culture medium every 24 h during the experiment and added the corresponding glucose to the initial culture concentration according to the results. Further study showed that the high glucose treatment significantly decreased the binding between Bad and Bcl-xL and Bad phosphorylation in the metformin group. In addition to our results, another study reported that metformin induced apoptosis by activating caspases 3/7 activity, decreasing Bcl-2 and Bcl-xl expression, and increasing Bax and Bad expression in epithelial ovarian cancer^41^. These results indicated that high glucose protects MSCs from metformin-induced apoptosis in an AMPK-mTOR-S6k1-Bad-dependent manner. However, the high expression of TNF-α due to long-term hyperglycemia and advanced glycation end products (AGEs) could decrease MSCs’ proliferation ability and increase MSC apoptosis via the caspase pathway and p38 MAPK pathway^42,43^. Thus, an appropriate glucose control target consideration both factors is urgently needed.

Differing from human MSCs based on the ISCT criteria, the criteria for mouse MSCs are highly heterogeneous. On the one hand, the markers of bone marrow derived mouse MSCs include the positive expression of CD29, CD44, CD105, CD73 and Sca-1 and the negative expression of CD11b, CD31, CD34, CD45, CD86, CD90 and MHC Class II^22,24,28^. On the other hand, the markers of other tissue (synovium, adult adipose tissue, epiphysis and lung) derived mouse MSCs include the extra positive expression including CD49e, CD90, CD106 and SSEA-4 and the extra negative expression including CD35, CD106, CD117, CD140a and MHC I^23,25–27^. Based on the above studies, the mBM-MSCs were characterized by CD45-CD105+ CD90+ Sca-1+ in this study. Expectedly, high glucose and compound C significantly reduced the metformin-induced mBM-MSC decrease.

Meta-analyses of previous randomized trials of glucose control and the association between glycated hemoglobin and macrovascular disease indicate that a 0.7% reduction in the glycated hemoglobin value might be expected to produce a reduction in the rate of macrovascular events by approximately one sixth^1,44^. However, recent evidence have shown that diabetes patients did not benefit from intensive glucose control in terms of cardiovascular complications and mortality as previously expected^7,8^. Moreover, based on the proportional hazards model between severe hypoglycemia and cardiovascular events, it was determined that a non-causal relationship exists between severe hypoglycemia and cardiovascular events^45^. Therefore, in addition to endogenous factors, such as the activation of the complement system in diabetic patients recently reported by our group^19^, intensive glucose control achieved by anti-diabetic drugs may have unexpected adverse effects, which could influence the outcomes of hypoglycemic therapy in diabetic patients. As expected, we found that high glucose inhibits metformin-induced MSC apoptosis in an AMPK-mTOR-S6K1-Bad-dependent manner. Our data help explain the lack of benefit associated with intensive glucose control in T_2_DM, which may be due to the exacerbated negative effects of metformin on MSCs during intensive glucose control therapy. Recent clinical trial data also support our theory that the underlying cause of these differences could be found in intensive glucose control; thus, the lower blood glucose level increases the adverse effects of metformin and leads to endogenous MSC apoptosis, which plays an important role in tissue repair and homeostasis.

Thus, the poorer clinical benefit of the intensive glucose control therapy strategy may be related to the adverse effects due to metformin-induced MSC apoptosis under intensive glucose control. It could be interesting to determine whether more moderate glucose control might provide long-term cardiac benefits in patients taking metformin. Consistent with our results, a recent clinical study showed that appropriate glucose control in the intensive care unit (ICU) reduces morbidity and overall mortality in critically ill patients^46^.

Our results highlight that high glucose could significantly attenuate the unexpected adverse effect of metformin-induced MSC apoptosis through AMPK-mediated mTOR suppression. Several new strategies can be considered, such as optimizing glucose control, taking metformin before a meal, or developing modified metformin with reduced adverse effects on MSCs.

## Supplementary information

Supplementary Information
