# Supplementary material for: Full title: High glucose protects mesenchymal stem cells from metformin-induced apoptosis through the AMPK-mediated mTOR pathway
Source: Sci Rep. 2019 Nov 28;9:17764. doi: 10.1038/s41598-019-54291-y (PMC6882892; doi:10.1038/s41598-019-54291-y)

Full title: High glucose protects mesenchymal stem cells from metformin-induced apoptosis through the AMPK-mediated mTOR pathway

Authors: Xiao He, Yi Yang, Meng-Wei Yao, Ting-ting Ren, Wei Guo, Ling Li, Xiang Xu

Supplementary information. The unprocessed images for Figure 2.

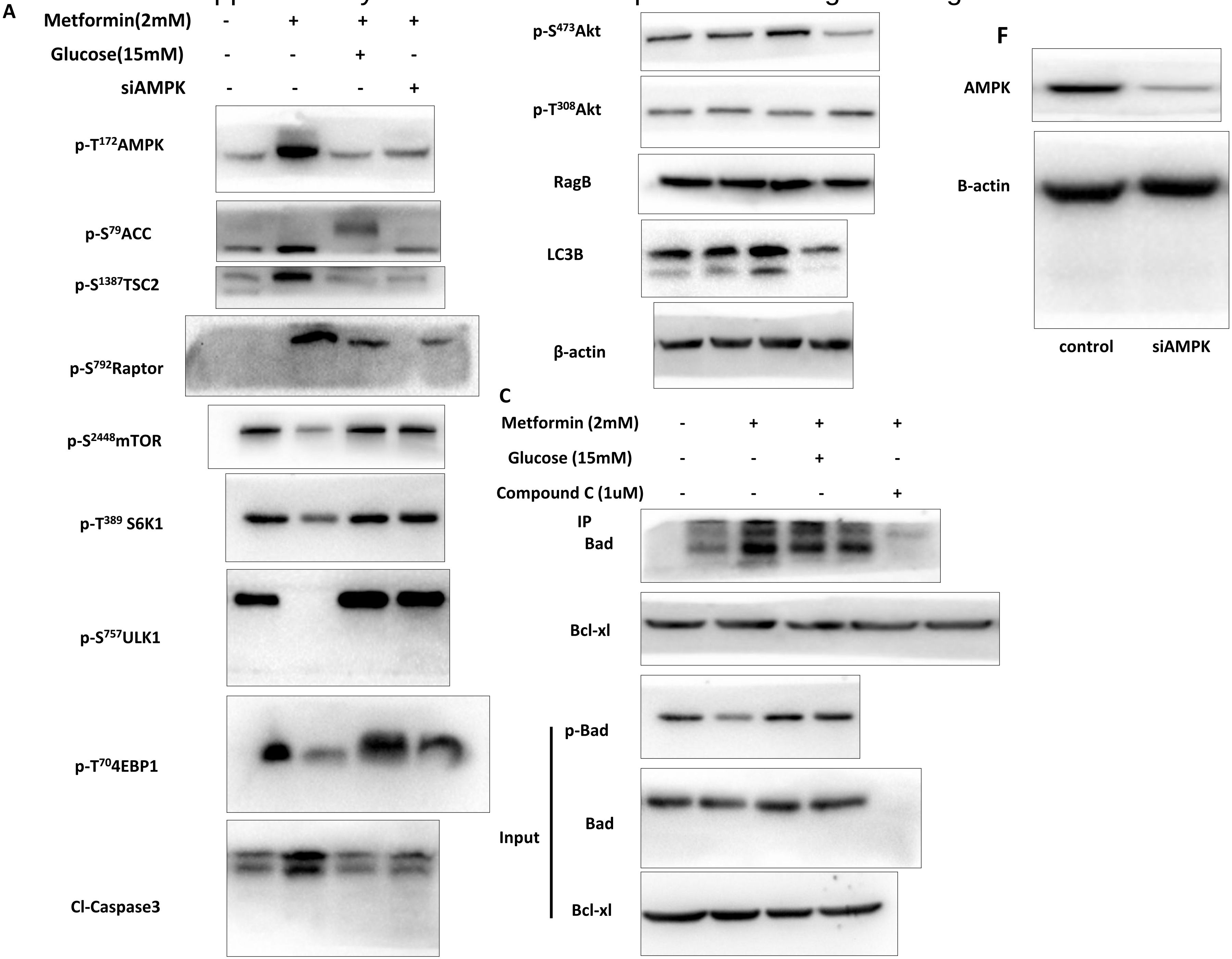

Supplement: Supplementary file 1 — Supplementary Information [file 41598_2019_54291_MOESM1_ESM.pdf]
